# ZBP1-mediated PANoptosis is a crucial lethal form in diverse keratinocyte death modalities in UVB-induced skin injury

**DOI:** 10.1038/s41419-025-07351-3

**Published:** 2025-01-26

**Authors:** Xuechan Bi, Min Li, Yiming Guo, Mengyao Hu, Yujie Chen, Ni Lian, Sihan Chen, Min Li, Heng Gu, Xu Chen

**Affiliations:** 1 Jiangsu Key Laboratory of Molecular Biology for Skin Diseases and STIs, Hospital for Skin Diseases, Institute of Dermatology, Chinese Academy of Medical Sciences & Peking Union Medical College, Nanjing, 210042 Jiangsu China; 2 State Key Laboratory of Natural Medicines, School of Basic Medicine and Clinical Pharmacy, China Pharmaceutical University, Nanjing, 211198 Jiangsu China; 3 School of Public Health, Nanjing Medical University, Nanjing, 211166 Jiangsu China

**Keywords:** Cell death, Mechanisms of disease, Acute inflammation

## Abstract

UVB irradiation induces diverse modalities of regulatory cell death in keratinocytes. Recently, the pattern of coexistence of pyroptosis, apoptosis, and necroptosis has been termed PANoptosis; however, whether PANoptosis occurs in keratinocytes in UVB-induced skin injury remains unclear. We observed that the key molecules of GSDMD-mediated pyroptosis, apoptosis, and necroptosis, which are N-terminal GSDMD, cleaved caspase-3/PARP, and phosphorylated MLKL, respectively, were elevated in keratinocytes of UVB-challenged mice and human skin tissue. Through keratinocyte-specific gene knockout or using corresponding inhibitors, we found that individual inhibition of GSDMD-mediated pyroptosis, caspase-3-mediated apoptosis, or MLKL-mediated necroptosis did not reduce the overall level of keratinocyte death after UVB exposure, and that the other two pathways maintained the activation. However, when the PANoptosome sensor ZBP1 was knocked out, keratinocyte death was reduced and epidermal thickening was alleviated in UVB-challenged mice. In conclusion, our study demonstrated that UVB irradiation induces ZBP1-mediated PANoptosis in keratinocytes, which is a crucial lethal form in diverse keratinocyte death modalities in UVB-induced skin injury. The above findings provide a new insight on the complexity of regulated cell death modalities in keratinocytes exposed to UV irradiation.

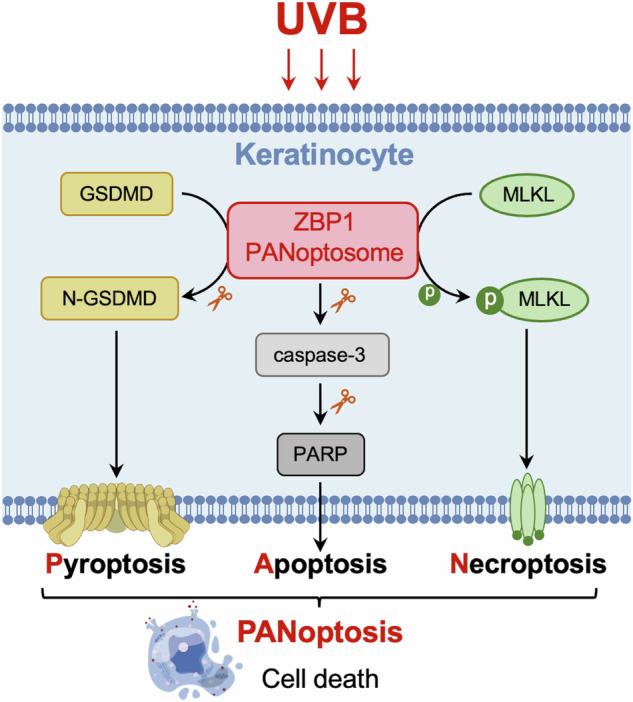

## Introduction

Prolonged exposure to ultraviolet (UV) of human skin can lead to UV-related skin diseases such as sunburn, photodermatoses, skin photoaging, precancerous lesions, and cancers [1]. The epidermis is the first barrier against UV irradiation, which absorbs the majority of UVB in UV. The high radiation intensity of UVB leads to a series of biological responses in keratinocytes of the epidermis, such as DNA damage [2], oxidative stress [3], aberrant production of cytokines [4] and damage-associated molecular patterns (DAMPs) [5], cell death [6] and so on. These abnormal responses of keratinocytes, especially UVB-induced keratinocyte death, are vital for the development of UVB-induced skin injury.

Recent studies have found that UVB-induced keratinocyte death exists in different forms of regulated cell death (RCD), such as apoptosis [7, 8] and ferroptosis [9, 10]. It has been proved that the levels of key molecules of apoptosis, cleaved PARP and cleaved caspase-3, were increased in HaCaT cells after UVB exposure [11]. Our group reported that keratinocytes in mice underwent GSDME-mediated pyroptosis after UVB exposure, along with the elevated expression of phosphorylated MLKL (p-MLKL) at Ser345, which is the key molecule of necroptosis [12]. In general, previous studies revealed that key molecules for GSDME-mediated pyroptosis, apoptosis, and necroptosis can be activated in keratinocytes after UVB irradiation.

The simultaneous occurrence pattern of pyroptosis, apoptosis, and necroptosis was named PANoptosis in 2019 [13], which is a complex inflammatory regulated cell death regulated by PANoptosome complex [14]. The above three RCD signals interact and regulate with each other extensively, and when one of these pathways is blocked, compensatory responses of other pathways may occur [15]. The typical pathway of pyroptosis in PANoptosis is mediated by GSDMD[16], which is different from the GSDME-mediated pyroptosis observed in our previous study [12]. PANoptosis has been found to play an important role in diseases such as infectious diseases, cancers, neurodegenerative diseases, and inflammatory diseases [17, 18]. Recently, Y. Saito et al. reported that PANoptosis is key to keratinocyte death in toxic epidermal necrolysis [19]. Similarly, UVB-induced skin injury also manifests as epidermal necrosis and inflammation in pathological features, but the role of PANoptosis in keratinocytes in UVB-induced skin injury remains unclear.

Above all, we aimed to figure out whether UVB irradiation can lead to PANoptosis in keratinocytes and explored the role of keratinocyte PANoptosis in UVB-induced skin injury.

## Materials and methods

### Reagents and antibodies

Disulfiram (HY-B0240) and Ac-DEVD-CHO (HY-P1001) were from MedChemExpress (Monmouth Junction, NJ, USA). Primary antibodies against PARP (#9532), cleaved PARP (#5625 for human), Caspase-3 (#9662), MLKL (#37705), phospho-MLKL (Ser345, #37333 for mouse), cleaved GSDMD (Asp275, #36425 for human), GSDMD (#39754), ZBP1 (#60968 for human), RIPK1 (#3493), RIPK3 (#95702), phospho-RIPK3 (Thr231/Ser232, #91702), Caspase-8 (#4927), AIM2 (#63660), ASC (#67824), F4/80 (#70076), β-Actin (#4970), GAPDH (#5174), and HRP-linked secondary antibodies, including anti-rabbit IgG (#7074) and anti-mouse IgG (#7076), were from Cell Signaling Technology (Danvers, MA, USA). Primary antibodies against phospho-MLKL (Ser358, #ab187091 for human), GSDMD (#ab209845 for mouse), NLRP3 (#ab263899), MPO (#ab208670), MMP-9 (#ab283575), CD4 (#ab183685), and CD8 (#ab217344) were from Abcam (Cambridge, MA, USA). Primary antibodies against ZBP1 (#sc-271483 for mouse), PYPAF7 (alias NLRP12, #sc-390666), and FADD (#sc-271748) were from Santa Cruz Biotechnology (Dallas, TX, USA). The primary antibody against cleaved caspase-1 (#PA5-105049) was from Invitrogen (Carlsbad, CA, USA). The primary antibody against ZBP1 (#13285-1-AP for human) was from Proteintech (Rosemont, IL, USA).

Among the above antibodies, cleaved PARP (#5625), cleaved GSDMD (Asp275, #36425), phospho-MLKL (Ser358, #ab187091), and ZBP1 (#13285-1-AP) were used for immunohistochemistry assays of human skin sections. MPO (#ab208670), MMP-9 (#ab283575), F4/80 (#70076), CD4 (#ab183685), and CD8 (#ab217344) were used for immunohistochemistry assays on mouse skin sections. The remaining antibodies were used for western blotting assay.

### Animal study

C57BL/6NGpt mice (wild type, WT) and *Zbp1*^−/−^ (*Zbp1* KO) mice were purchased from GemPharmatech (Nanjing, Jiangsu, China). Keratinocyte-specific *Gsdmd* conditional knockout (cKO) mice (Krt14^Cre/+^-*Gsdmd*^flox/flox^), keratinocyte-specific *Mlkl* cKO mice (Krt14^Cre/+^-*Mlkl*^flox/flox^) and their littermate control mice (Krt14^+/+^-*Gsdmd*^flox/flox^ and Krt14^+/+^-*Mlkl*^flox/flox^) were obtained from GemPharmatech as previously described [12, 20].

All the mice used were 6–10 weeks of age and 15–30 g of weight, including males and females. Mice were randomly assigned to experimental groups. We shaved the mice dorsal skin 24 h before UVB irradiation. To establish an acute UVB injury model, mice were exposed to low-dose (215 mJ/cm^2^) or high-dose (430 mJ/cm^2^) of UVB [21] by UVB lamps (Philips, UVB Broadband PL-S 9 W/12). The scores of UVB damage were used to evaluate the level of UVB skin damage as previously described [12]. Two investigators blinded to study subgroups scored the severity of skin lesions in all groups. All mice were observed and sacrificed at the designated times after UVB exposure. Dorsal skin samples were collected and immersed in Dispase II (2 mg/ml, Sigma-Aldrich, St. Louis, MO, USA) at 4 °C for 17 h to separate the epidermis.

Animal studies were approved by the Medical Ethics Committee in Institute of Dermatology, Chinese Academy of Medical Sciences (Approval Number: 2022-DW-018).

### Preparation and application of medications in animal studies

The preparation and application of disulfiram solution and Ac-DEVD-CHO solution are described in Table 1 according to previous studies [22, 23] and manufacturer’s instructions. The corresponding vehicle without active pharmaceutical ingredient was prepared at the same time as the control.

**Table 1:** The preparation of medications in animal studies.

Medication	Working solution concentration	Other ingredients	Dose and application each day per mouse
Disulfiram	5 mg/ml	10% DMSO 40% PEG400 5% Tween-80 45% Saline	50 mg/kg for intraperitoneal injection
Ac-DEVD-CHO	0.3 mg/ml	PBS	3 mg/kg for intraperitoneal injection

### Human skin samples

12 human skin samples from sun-exposed area (face), 12 skin samples from non-exposed areas (abdomen, hip, and inner thigh), and 3 healthy skin samples were obtained from the biobank of Institute of Dermatology, Chinese Academy of Medical Sciences, Jiangsu Biobank of Clinical Resources (BM2015004). Informed consent was obtained from all subjects. This study was approved by the Medical Ethics Committee in Institute of Dermatology, Chinese Academy of Medical Sciences (Approval Number: 2022-KY-057).

### Human skin explants and UVB radiation

Upon separation from the human body, the healthy skin sample was washed with 1× Penicillin Streptomycin Glutamine (Gibco, Invitrogen Corp., Carlsbad, CA, USA) immediately in a biological cabinet. After removing as much subcutaneous fat issue as possible with scissors, the skin sample was divided into two halves. One was for acute UVB exposure, and another one served as a control. The divided skin tissue was padded with small pieces of filter paper in a culture dish, and EpiLife™ CF Medium (Gibco, Invitrogen Corp., Carlsbad, CA, USA) was added to saturate the filter paper. Human skin explants were cultured at 37 °C in a CO_2_ incubator.

To establish an acute UVB injury model ex vivo, human skin explants were exposed to 600 mJ/cm^2^ UVB [24, 25] by UVB lamps (Philips, UVB Broadband PL-S 9 W/12). The samples for hematoxylin and eosin (H&E) staining and western blotting assay were prepared from skin explants at 48 h after UVB exposure. The samples for western blotting assay were immersed in Dispase II (2 mg/ml, Sigma-Aldrich) at 4 °C for 17 h to isolate the epidermis.

### Histological and immunohistochemistry analysis

H&E staining and immunohistochemistry assays were performed as previously described [26]. Epidermal thickness was measured in three randomly selected fields of view of non-necrotic epidermis in H&E-stained sections, and the values were averaged to assess the degree of epidermal edema and hyperplasia. The average optical density (AOD) in immunohistochemical staining of terminal deoxynucleotidyl transferase-mediated dUTP nick end labeling (TUNEL), MPO, MMP9, CD4, CD8, F4/80, N-terminal GSDMD (N-GSDMD), cleaved PARP, p-MLKL, and ZBP1 was calculated by ImageJ software (National Institutes of Health, USA) in three randomly selected fields of view with 40× magnification, and their mean values were used for statistical analysis.

### Cell culture and UVB radiation

Human primary keratinocytes from ATCC (PCS-200-010) were cultured in Dermal Cell Basal Medium (ATCC PCS-200-030) containing Keratinocyte Growth Kit (ATCC PCS-200-040). Human primary keratinocytes were exposed to 50 mJ/cm^2^ UVB by UVB lamps (Philips, UVB Broadband PL-S 9 W/12) and collected after 12 h of UVB irradiation.

### Western blotting assay

RIPA lysis buffer (P0013B, Beyotime Biotechnology, Shanghai, China) containing protease and phosphatase inhibitors (Roche Applied Science, Basel, Switzerland) was used to lyse human primary keratinocytes and the epidermis isolated from mice skin and human skin explants. Western blotting assay was performed as previously described [26]. The density of the interested protein band was calculated using ImageJ software and compared with β-actin (ACTB) as relative quantification.

### Quantitative reverse transcription PCR (qRT-PCR)

Total RNA extraction, RNA reverse transcription, and quantitative real-time PCR were performed using RNAex Pro Reagent, Evo M-MLVRT kit, SYBR Green Premix Pro Taq HS qPCR Kit (all from Accurate Biotechnology Co., Ltd, Changsha, China) and Light-Cycler 480 (Roche, Basel, Switzerland) according to the manufacturers’ instructions. Primer sequences provided by GemPharmatech are listed in Table 2. The relative mRNA level was calculated by 2^−∆∆Ct^ method.

**Table 2:** Primer sequences in this study.

Primer	Sequences (5’ → 3’)
Zbp1-m-F	ACCTTCTGAGCTATGACGGACAGAC
Zbp1-m-R	GGCGTTTGAATTGGCAATGGAG
Actin-m-F	GGCTGTATTCCCCTCCATCG
Actin-m-R	CCAGTTGGTAACAATGCCATGT

### Statistical analysis

We obtained data for statistical analysis from at least three independent experiments. All data were shown as mean ± standard deviation (SD). Student’s *t* test or adjusted t-test was used to analyze the difference between two groups. One-way ANOVA or Kruskal–Wallis test was used to analyze the differences among three or four groups. Data analysis was performed with SPSS Statistics software (IBM Corp., USA). P < 0.05 was considered statistically significant.

## Results

### UVB irradiation simultaneously upregulated indicators of pyroptosis, apoptosis and necroptosis in mice keratinocytes

**Fig. 1:**
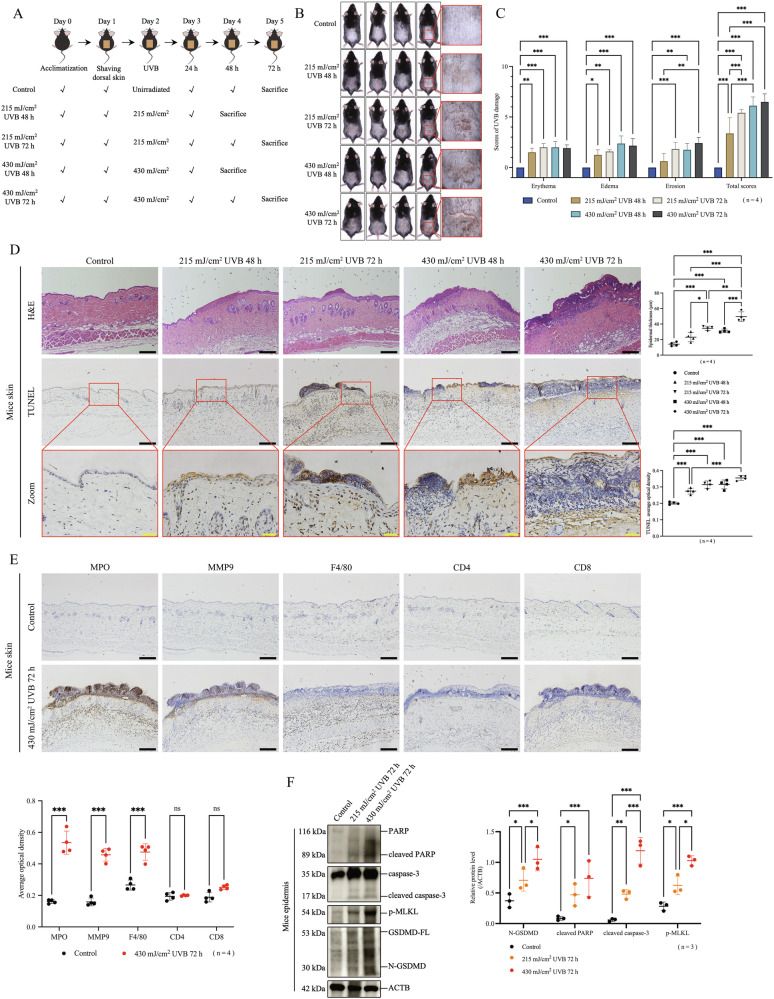
UVB irradiation simultaneously upregulated indicators of pyroptosis, apoptosis and necroptosis in mice keratinocytes. **A** Flowchart of WT mice exposed or not exposed to low-dose (215 mJ/cm^2^) or high-dose (430 mJ/cm^2^) of UVB irradiation, and sacrificed at 48 or 72 h after UVB irradiation. **B** Photos of mice dorsal skin after different UVB irradiation doses and times (n = 4). **C** The scores of UVB damage of mice skin after different UVB irradiation doses and times (n = 4). **D** Representative images of H&E staining and TUNEL staining of mice skin sections after different UVB irradiation doses and times. The statistic of epidermal thickness and average optical density of TUNEL positive staining were analyzed (n = 4). **E** Representative images of MPO, MMP9, F4/80, CD4, and CD8 immunohistochemical staining in control mice and 430 mJ/cm^2^ UVB-irradiated mice at 72 h. The statistic of average optical densities of MPO, MMP9, F4/80, CD4, and CD8 positive staining were analyzed (n = 4). **F** Proteins of N-GSDMD, cleaved PARP, cleaved caspase-3, and p-MLKL were detected by western blotting assay in epidermal lysates of control, 215 mJ/cm^2^ UVB 72 h, and 430 mJ/cm^2^ UVB 72 h groups of mice (sacrificed on the same day). The relative protein levels of the above proteins were compared with ACTB as quantification (n = 3). The scale bar in black is 100 μm for 10× magnification in **D** and **E**. The scale bar in yellow is 20 μm for 40× magnification in **D**. *P < 0.05. **P < 0.01. ***P < 0.001. ns not significant. GSDMD-FL GSDMD full length. N-GSDMD N-terminal GSDMD. p-MLKL phosphorylated MLKL.

We established acute UVB injury model on mice dorsal skin and found that the severity of erythema, edema, and erosion were positively correlated with UVB dose (Fig. 1A–C). Similar results were observed in histological features; with increasing irradiation dose, the epidermal gradually thickened, the degree of epidermal necrosis and inflammatory cell infiltration gradually intensified, and the TUNEL positive staining in epidermis gradually increased (Fig. 1D). Results of immunohistochemistry assay showed that at 72 h after high-dose (430 mJ/cm^2^) UVB irradiation, positive staining of neutrophil markers (MPO, MMP9) and macrophage marker (F4/80) were increased compared with that of unirradiated, whereas positive staining of T cell markers (CD4, CD8) showed no obvious difference between two groups (Fig. 1E). The above results suggest that UVB irradiation induced an increase in epidermal cell death and infiltration of immune cells including neutrophils and macrophages after UVB irradiation. Importantly, by western blotting assay, we found that the expressions of N-GSDMD, cleaved PARP, cleaved caspase-3, and p-MLKL were all elevated in mice epidermis at 72 h after UVB irradiation (Fig. 1F). These results indicate that UVB irradiation simultaneously upregulates molecular indicators of pyroptosis, apoptosis, and necroptosis in mouse keratinocytes, indicating that PANoptosis may be triggered by UVB irradiation.

### UVB irradiation simultaneously upregulated indicators of pyroptosis, apoptosis and necroptosis in human keratinocytes

**Fig. 2:**
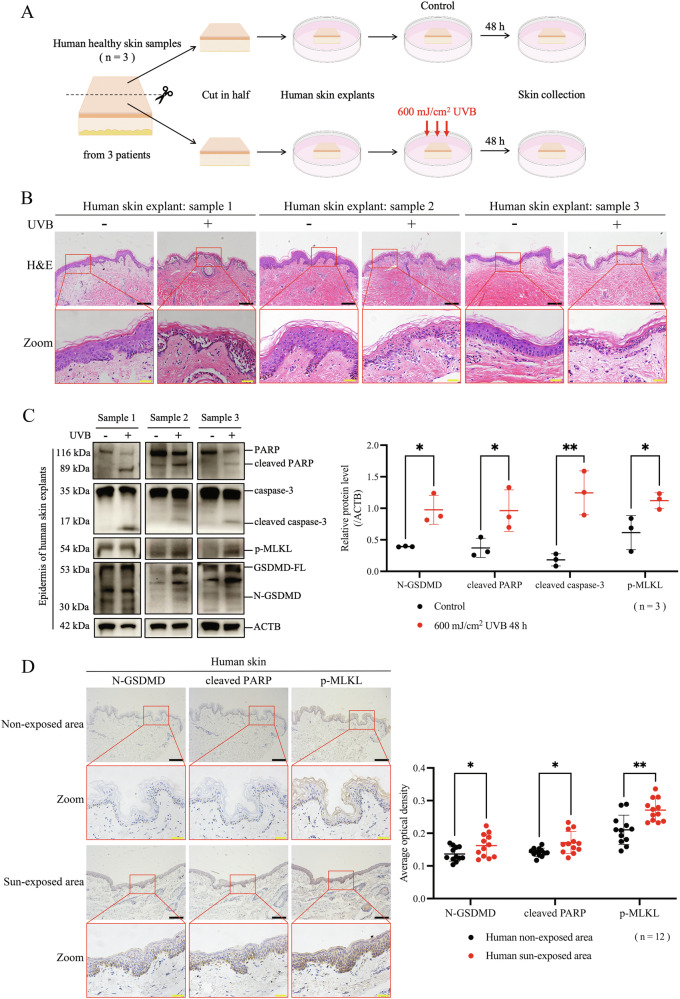
UVB irradiation simultaneously upregulated indicators of pyroptosis, apoptosis and necroptosis in human keratinocytes. **A** Flowchart of acute UVB injury model in human skin explants. **B** Images of H&E staining of human skin explants from 3 samples. **C** Proteins of N-GSDMD, cleaved PARP, cleaved caspase-3, and p-MLKL were detected by western blotting assay in epidermal lysates of human skin explants from 3 samples. The relative protein levels of the above proteins were compared with ACTB as quantification (n = 3). **D** Representative images of immunohistochemical staining of N-GSDMD, cleaved PARP, and p-MLKL in human skin samples from sun-exposed and non-exposed areas. The average optical densities of N-GSDMD, cleaved PARP, and p-MLKL positive staining were statistically analyzed (n = 12). The scale bar in black is 100 μm for 10× magnification in **B** and **D**. The scale bar in yellow is 20 μm for 40× magnification in **B** and **D**. *P < 0.05. **P < 0.01. ***P < 0.001. ns not significant. GSDMD-FL GSDMD full length. N-GSDMD N-terminal GSDMD. p-MLKL phosphorylated MLKL.

To verify whether the above effects also occur in human skin tissue, we used human skin explants to construct an acute UVB injury model ex vivo (Fig. 2A). We found that at 48 h after 600 mJ/cm^2^ UVB irradiation, normal cellular structures within the epidermis were severely destroyed, manifesting abnormal cellular staining, irregular nuclear morphology, and cytoplasmic vacuolation (Fig. 2B), indicating increased keratinocyte death. Importantly, the levels of N-GSDMD, cleaved PARP, cleaved caspase-3 and p-MLKL were all elevated in the epidermis after UVB irradiation by western blotting assay (Fig. 2C). These results suggest that, accompanied with occurrence of keratinocyte death, PANoptosis indicators were detected in human skin tissue with acute UVB injury.

We further validated the occurrence of PANoptosis indicators in human skin with chronic UV injury. Human skin samples of sun-exposed and non-exposed areas were collected and performed immunohistochemical studies for N-GSDMD, cleaved PARP, and p-MLKL. We found that the levels of the above three indicators in the epidermis of sun-exposed skin were higher than those of non-exposed skin areas (Fig. 2D). Linear regression analysis revealed a positive correlation between the expression levels of N-GSDMD and cleaved PARP in human sun-exposed skin, and age was also positively related with the levels of N-GSDMD and cleaved PARP (P < 0.05) (Table 3). In non-exposed human skin, N-GSDMD, cleaved PARP, and p-MLKL were positively correlated with each other, meanwhile N-GSDMD and p-MLKL were positively correlated with age (P < 0.05) (Table 3). These findings suggest that the indicators of PANoptosis simultaneously occur in the epidermis of human skin.

**Table 3:** Linear regression analysis of N-GSDMD, cleaved PARP, p-MLKL and age.

			N-GSDMD	cleaved PARP	p-MLKL	Age
Human non-exposed area	Pearson’s r	N-GSDMD	1	0.672	0.554	0.557
		cleaved PARP	0.672	1	0.547	0.476
		p-MLKL	0.554	0.547	1	0.677
		Age	0.557	0.476	0.677	1
	*P* (one-tailed)	N-GSDMD	–	0.008	0.031	0.03
		cleaved PARP	0.008	–	0.033	0.059
		p-MLKL	0.031	0.033	–	0.008
		Age	0.03	0.059	0.008	–
Human sun-exposed area	Pearson’s r	N-GSDMD	1	0.716	−0.048	0.595
		cleaved PARP	0.716	1	−0.132	0.717
		p-MLKL	−0.048	−0.132	1	−0.1
		Age	0.595	0.717	-0.1	1
	*P* (one-tailed)	N-GSDMD	–	0.004	0.441	0.021
		cleaved PARP	0.004	–	0.342	0.004
		p-MLKL	0.441	0.342	–	0.378
		Age	0.021	0.004	0.378	–

### Blocking GSDMD-mediated pyroptosis alone did not decrease UVB-induced keratinocyte death

**Fig. 3:**
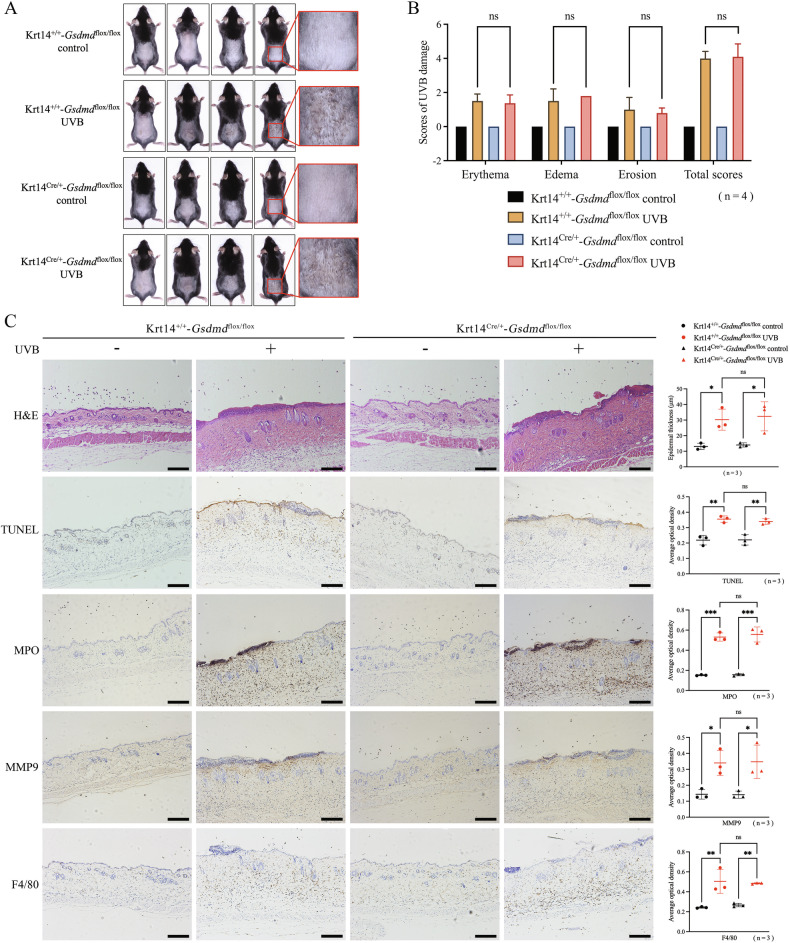

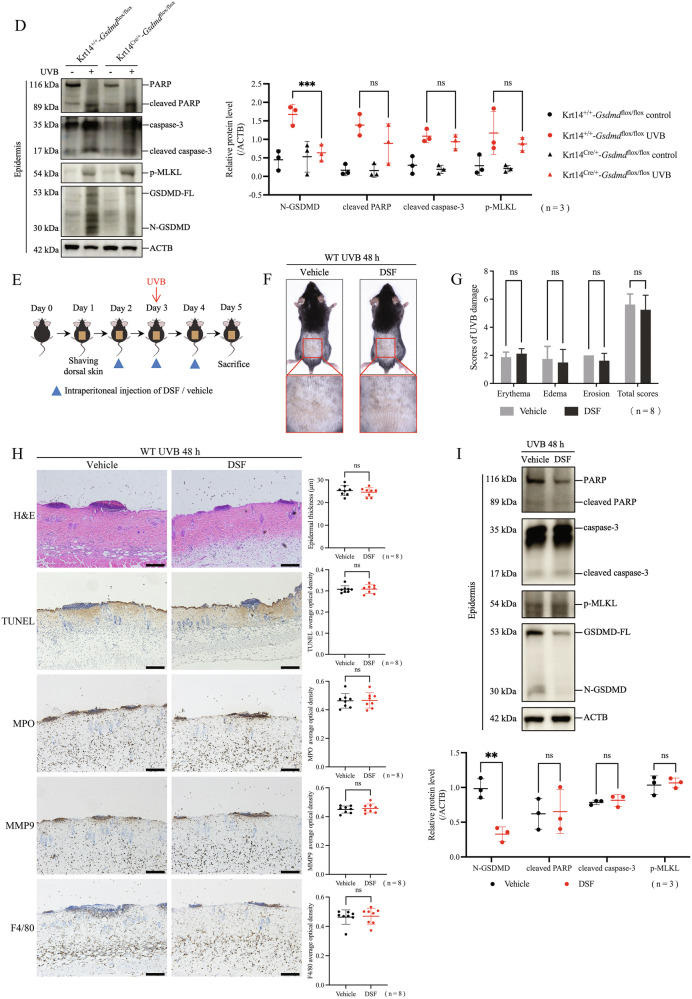
Blocking GSDMD-mediated pyroptosis alone did not decrease UVB-induced keratinocyte death. **A** Photos of keratinocyte-specific *Gsdmd* cKO mice (Krt14^Cre/+^-*Gsdmd*^flox/flox^) and control mice (Krt14^+/+^-*Gsdmd*^flox/flox^) at 48 h after exposure or non-exposure to 430 mJ/cm^2^ UVB irradiation (n = 4). **B** UVB skin damage of Krt14^Cre/+^-*Gsdmd*^flox/flox^ mice and Krt14^+/+^-*Gsdmd*^flox/flox^ mice was evaluated by scores (n = 4). **C** Representative images of H&E, TUNEL, MPO, MMP9, F4/80 staining in Krt14^Cre/+^-*Gsdmd*^flox/flox^ mice and Krt14^+/+^-*Gsdmd*^flox/flox^ mice after exposure or non-exposure to UVB irradiation. The statistic of epidermal thickness and average optical densities of TUNEL, MPO, MMP9, F4/80 positive staining were analyzed (n = 3). **D** Proteins of N-GSDMD, cleaved PARP, cleaved caspase-3, and p-MLKL in the epidermis of Krt14^Cre/+^-*Gsdmd*^flox/flox^ mice and Krt14^+/+^-*Gsdmd*^flox/flox^ mice were detected by western blotting assay. The relative protein levels of the interested proteins were compared with ACTB as quantification (n = 3). **E** Flowchart of DSF treatment or vehicle treatment for acute UVB skin injury model in WT mice (48 h after 430 mJ/cm^2^ UVB irradiation). **F** Representative photos of mice receiving DSF or vehicle treatment at 48 h after UVB exposure. **G** UVB skin damage of DSF or vehicle treated mice was evaluated by scores (n = 8). **H** Representative images of H&E, TUNEL, MPO, MMP9, and F4/80 staining in DSF or vehicle treated mice after UVB irradiation. Statistical analysis of epidermal thickness and average optical densities of TUNEL, MPO, MMP9, F4/80 positive staining were shown (n = 8). **I** Proteins of GSDMD-FL, N-GSDMD, cleaved PARP, cleaved caspase-3, and p-MLKL in the epidermis of DSF or vehicle treated mice were detected by western blotting assay. The relative protein levels of the interested proteins were compared with ACTB as quantification (n = 3). The scale bar in black is 100 μm for 10× magnification in **C** and **H**. *P < 0.05. **P < 0.01. ***P < 0.001. ns not significant. DSF disulfiram. GSDMD-FL GSDMD full length. N-GSDMD N-terminal GSDMD. p-MLKL phosphorylated MLKL.

To elucidate the dominant form of RCDs in keratinocytes during UVB injury, we first investigated the role of GSDMD-mediated pyroptosis in this process. We chose 48 h after 430 mJ/cm^2^ UVB irradiation as the observation time point. The reason is that the mice already showed obvious skin lesions and cell death at this time point according to Fig. 1B–D. We found no significant differences in skin erythema, edema, or erosion scores between keratinocyte-specific *Gsdmd* cKO mice and littermate control mice at 48 h after UVB irradiation (Fig. 3A, B). We also did not observe differences in epidermal thickness (H&E staining), keratinocyte death levels (TUNEL staining), infiltration of immune cells such as neutrophils (MPO and MMP9 staining) or macrophages (F4/80 staining) between two groups of mice after UVB irradiation (Fig. 3C). By western blotting assay, we verified that GSDMD was expressed at a low level in keratinocyte-specific *Gsdmd* cKO mice, and that cleaved PARP, cleaved caspase-3, and p-MLKL were still expressed at high levels in *Gsdmd* cKO mice after UVB irradiation, which levels were similar to those in littermate control mice (Fig. 3D).

To further validate the above results, we used disulfiram (DSF), a GSDMD inhibitor that can prevent GSDMD pore formation and pyroptosis [27]. We did not observe significant differences in UVB skin damage scores, epidermal thickness, keratinocyte death levels, or infiltration of neutrophils or macrophages between DSF treatment group and vehicle treatment group after UVB exposure (Fig. 3E–H). Compared with vehicle treatment group, DSF treatment decreased the levels of GSDMD and N-GSDMD, without affecting the levels of apoptosis proteins (cleaved PARP and cleaved caspase-3) or necroptosis protein (p-MLKL) after UVB exposure, as shown by western blotting assay (Fig. 3I). These results suggest that blocking GSDMD-mediated pyroptosis alone in keratinocytes cannot reduce UVB-induced keratinocyte death and skin damage in mice.

### Inhibiting caspase-3-mediated apoptosis alone did not decrease UVB-induced keratinocyte death

**Fig. 4:**
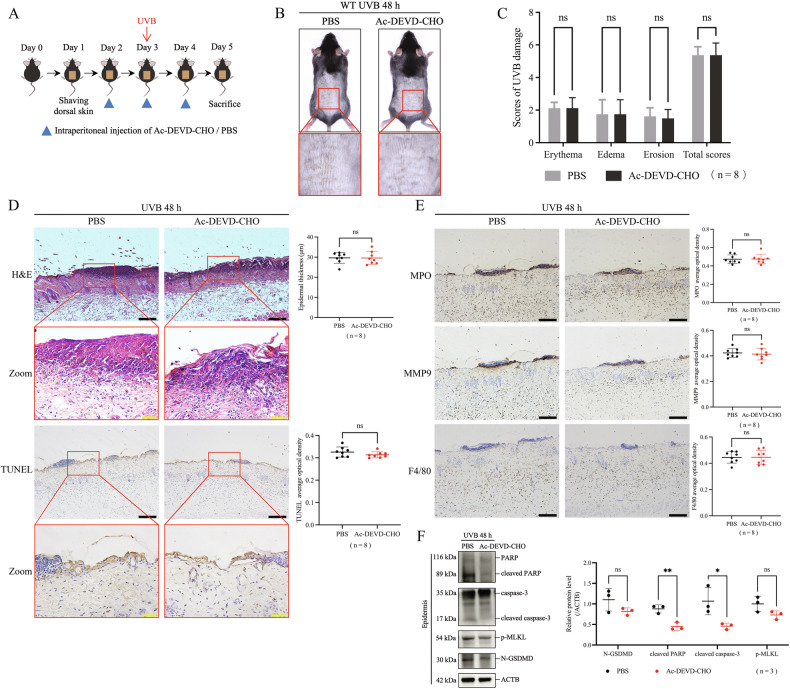
Inhibiting caspase-3-mediated apoptosis alone did not decrease UVB-induced keratinocyte death. **A** Flowchart of Ac-DEVD-CHO treatment or PBS treatment for acute UVB skin injury model in WT mice (48 h after 430 mJ/cm^2^ UVB). **B** Representative photos of mice receiving Ac-DEVD-CHO or PBS treatment 48 h after UVB exposure. **C** UVB skin damage of Ac-DEVD-CHO or PBS treated mice was evaluated by scores (n = 8). **D** Representative images of H&E staining and TUNEL staining of skin sections from Ac-DEVD-CHO or PBS treated mice after UVB irradiation. The statistic of epidermal thickness and average optical density of TUNEL positive staining were analyzed (n = 8). **E** Representative images of MPO, MMP9, and F4/80 immunohistochemical staining in Ac-DEVD-CHO or PBS treated mice after UVB irradiation. The statistic of average optical densities of MPO, MMP9, F4/80 positive staining were analyzed (n = 8). **F** Proteins of N-GSDMD, cleaved PARP, cleaved caspase-3, and p-MLKL in the epidermis of Ac-DEVD-CHO or PBS treated mice were detected by western blotting assay. The relative protein levels of the above proteins were compared with ACTB as quantification (n = 3). The scale bar in black is 100 μm for 10× magnification in **D** and **E**. The scale bar in yellow is 20 μm for 40× magnification in **D**. *P < 0.05. **P < 0.01. ns not significant. N-GSDMD N-terminal GSDMD. p-MLKL phosphorylated MLKL.

To block UVB-induced apoptosis, we used the caspase-3 inhibitor Ac-DEVD-CHO [22] to observe the effects (Fig. 4A). We found that Ac-DEVD-CHO-treated mice showed no significant difference in the scores of skin erythema, edema, and erosion after UVB irradiation compared to PBS-treated mice after UVB exposure (Fig. 4B, C). Epidermal thickness and epidermal necrosis did not differ between Ac-DEVD-CHO-treated mice and PBS-treated mice according to H&E staining assay (Fig. 4D). The overall level of UVB-induced keratinocyte death was not reduced after Ac-DEVD-CHO treatment by TUNEL staining assay (Fig. 4D). The positive staining of MPO, MMP9, and F4/80 showed no differences between Ac-DEVD-CHO-treated mice and PBS-treated mice, indicating similar levels of neutrophils and macrophages infiltration in both groups (Fig. 4E). By western blotting assay, we verified that the cleavage of PARP and caspase-3 by UVB exposure were inhibited in the epidermis after Ac-DEVD-CHO injection, whereas N-GSDMD and p-MLKL were not affected (Fig. 4F). These results suggest that inhibiting caspase-3-mediated apoptosis alone cannot alleviate UVB-induced keratinocyte death and skin damage in mice.

### Blocking MLKL-mediated necroptosis alone did not decrease UVB-induced keratinocyte death

**Fig. 5:**
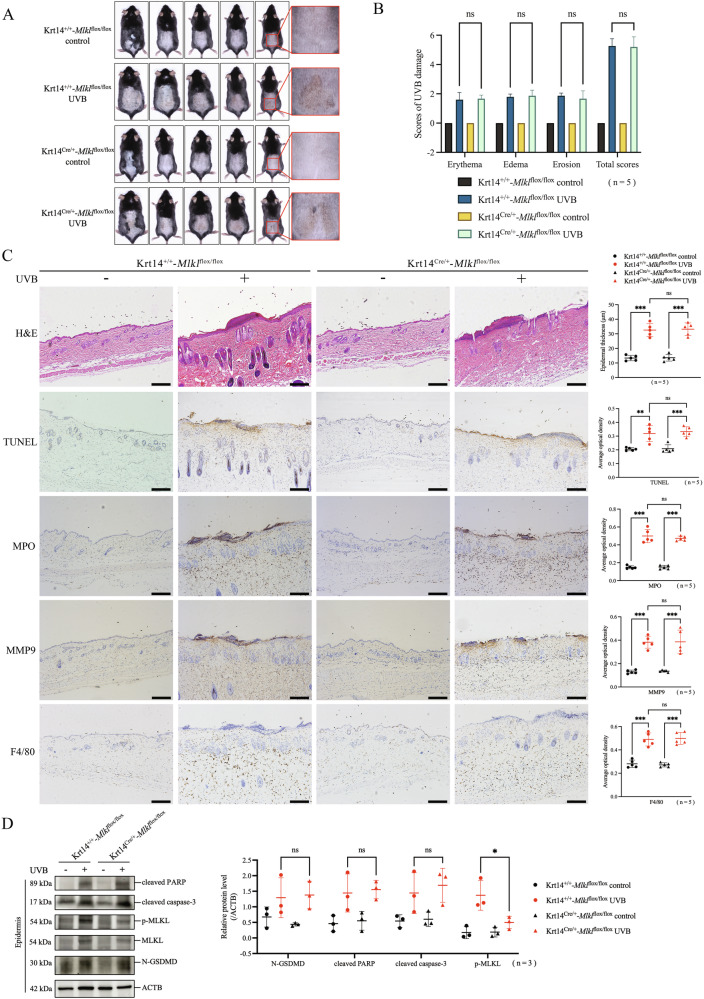
Blocking MLKL-mediated necroptosis alone did not decrease UVB-induced keratinocyte death. **A** Photos of keratinocyte-specific *Mlkl* cKO mice (Krt14^Cre/+^-*Mlkl*^flox/flox^) and control mice (Krt14^+/+^-*Mlkl*^flox/flox^) at 48 h after exposure or non-exposure to 430 mJ/cm^2^ UVB irradiation (n = 5). **B** UVB skin damage of Krt14^Cre/+^-*Mlkl*^flox/flox^ mice and Krt14^+/+^-*Mlkl*^flox/flox^ mice was evaluated by scores (n = 5). **C** Representative images of H&E, TUNEL, MPO, MMP9, and F4/80 staining in Krt14^Cre/+^-*Mlkl*^flox/flox^ mice and Krt14^+/+^-*Mlkl*^flox/flox^ mice after exposure or non-exposure to UVB irradiation. Statistical analysis of epidermal thickness and average optical densities of TUNEL, MPO, MMP9, F4/80 positive staining were shown (n = 5). **D** Proteins of N-GSDMD, cleaved PARP, cleaved caspase-3, MLKL and p-MLKL in the epidermis of Krt14^Cre/+^-*Mlkl*^flox/flox^ mice and Krt14^+/+^-*Mlkl*^flox/flox^ mice were detected by western blotting assay. The relative protein levels of the interested proteins were compared with ACTB as quantification (n = 3). The scale bar is 100 μm in **C**. *P < 0.05. **P < 0.01. ***P < 0.001. ns not significant. N-GSDMD N-terminal GSDMD. p-MLKL phosphorylated MLKL.

We proceeded to investigate the role of MLKL-mediated necroptosis of keratinocytes in UVB injury. We found that there were no significant differences in UVB skin damage scores, epidermal thickness, keratinocyte death levels, or neutrophils or macrophages infiltration between keratinocyte-specific *Mlkl* cKO mice and littermate control mice at 48 h after UVB irradiation (Fig. 5A–C). Although necroptosis key molecule (p-MLKL) was expressed at a low level in keratinocyte-specific *Mlkl* cKO mice by western blotting assay, pyroptosis key molecule (N-GSDMD) and apoptosis key molecules (cleaved PARP and cleaved caspase-3) remained highly expressed after UVB irradiation (Fig. 5D). These findings demonstrate that blocking MLKL-mediated necroptosis alone in keratinocytes still does not mitigate UVB-induced keratinocyte death and skin damage.

### ZBP1 is a key molecule in UVB-induced keratinocyte PANoptosis

**Fig. 6:**
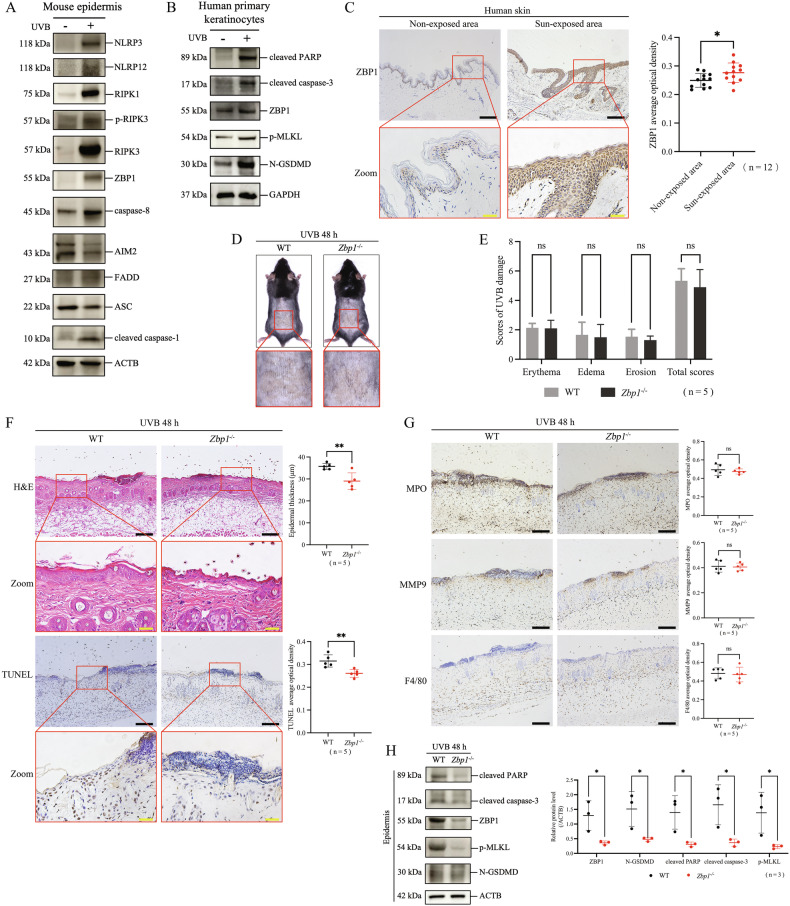
ZBP1 is a key molecule in UVB-induced keratinocyte PANoptosis. **A** Constitutive proteins related to PANoptosome including ZBP1, AIM2, NLRP3, NLRP12, RIPK1, RIPK3, p-RIPK3, caspase-8, cleaved caspase-1, FADD, and ASC in mice epidermal lysates were detected by western blotting assay at 48 h after 430 mJ/cm^2^ UVB irradiation. **B** Proteins of N-GSDMD, cleaved PARP, cleaved caspase-3, p-MLKL, and ZBP1 in human primary keratinocytes were detected by western blotting assay after UVB irradiation. **C** Representative images of immunohistochemical staining of ZBP1 in human skin samples from sun-exposed and non-exposed areas. Statistical analysis of average optical density of ZBP1 positive staining was shown (n = 12). **D** Representative photos of WT mice and *Zbp1*^−/−^ mice at 48 h after 430 mJ/cm^2^ UVB exposure. **E** UVB skin damage of WT mice and *Zbp1*^−/−^ mice was evaluated by scores (n = 5). **F** Representative images of H&E staining and TUNEL staining of skin sections from WT mice and *Zbp1*^−/−^ mice after UVB irradiation. The statistic of epidermal thickness and average optical density of TUNEL positive staining were analyzed (n = 5). **G** Representative images of MPO, MMP9, and F4/80 immunohistochemical staining in WT mice and *Zbp1*^−/−^ mice after UVB irradiation. The statistic of average optical densities of MPO, MMP9, and F4/80 positive staining were analyzed (n = 5). **H** Proteins of ZBP1, N-GSDMD, cleaved PARP, cleaved caspase-3, and p-MLKL in the epidermis of WT mice and *Zbp1*^−/−^ mice were detected by western blotting assay. The relative protein levels of the above proteins were compared with ACTB as quantification (n = 3). The scale bar in black is 100 μm for 10× magnification in **C**, **F**, and **G**. The scale bar in yellow is 20 μm for 40× magnification in **C** and **F**. *P < 0.05. **P < 0.01. ns not significant. N-GSDMD N-terminal GSDMD. p-MLKL phosphorylated MLKL.

Since blocking any pathway of pyroptosis, apoptosis, or necroptosis alone cannot affect the overall level of UVB-induced keratinocyte death, combined with the simultaneous occurrence of three pathways indicators in UVB injury described in Fig. 1, we hypothesized that PANoptosis might play a crucial role in UVB-induced skin injury. PANoptosis is modulated by PANoptosome complex, so we first detected the changes of some constitutive proteins related to PANoptosome [28, 29] in mice epidermal lysates at 48 h after 430 mJ/cm^2^ UVB irradiation via western blotting. We found that UVB irradiation upregulated the protein levels of ZBP1, NLRP3, NLRP12, RIPK1, RIPK3, p-RIPK3, caspase-8, cleaved caspase-1 and FADD, while downregulating the levels of AIM2 and ASC (Fig. 6A). These findings suggest that UVB irradiation can lead to an obvious modulatory effect on PANoptosome in keratinocytes.

ZBP1 is one of the identified key sensors for initiation and assembly of PANoptosome; therefore, we explored whether ZBP1 is involved in UVB-induced keratinocyte PANoptosis. In human primary keratinocytes, accompanied with UVB-induced upregulation of N-GSDMD, cleaved-PARP, cleaved caspase-3, and p-MLKL, the level of ZBP1 was also elevated (Fig. 6B). Moreover, we found that ZBP1 was higher expressed in the epidermis of sun-exposed human skin than in that of non-exposed human skin (same samples in Fig. 1G) according to the results of ZBP1 immunohistochemical staining assay (Fig. 6C).

To clarify whether ZBP1 is a sensor of UVB-induced PANoptosis in keratinocytes, we observed skin damage and molecular indicators of PANoptosis in *Zbp1*^−/−^ mice after UVB irradiation. We verified that ZBP1 was knocked out in *Zbp1*^−/−^ mice using qRT-PCR (Supplementary Fig. S1). We found no significant differences in skin erythema, edema, or erosion between *Zbp1*^−/−^ mice and WT mice at 48 h after UVB irradiation (Fig. 6D, E). Importantly, UVB-induced epidermal thickening and keratinocyte death were alleviated in *Zbp1*^−/−^ mice compared with WT mice according to the results of H&E staining and TUNEL staining assay (Fig. 6F). Nevertheless, the degrees of neutrophil and macrophage infiltration were similar in *Zbp1*^−/−^ mice and WT mice after UVB irradiation (Fig. 6G). We verified that ZBP1 protein was expressed at a low level in epidermal lysates of *Zbp1*^−/−^ mice by western blotting assay. Compared with WT mice, the protein levels of N-GSDMD, cleaved PARP, cleaved caspase-3, and p-MLKL decreased in *Zbp1*^−/−^ mice after UVB irradiation (Fig. 6H). These results demonstrate that the knockout of ZBP1 can inhibit the simultaneous occurrence of UVB-induced keratinocyte PANoptosis indicators and keratinocyte death. However, ZBP1 knockout still did not alleviate inflammatory immune cell infiltration.

Taken together, our study confirmed that UVB irradiation can induce ZBP1-mediated PANoptosis in keratinocytes, which is a crucial lethal form in complex keratinocyte death modalities in UVB-induced skin injury.

## Discussion

In this study, we provide a novel insight on the complexity of UVB-induced regulated cell death in keratinocytes. UVB irradiation induces ZBP1-mediated PANoptosis in keratinocytes, which is a crucial lethal form of keratinocyte death in UVB-induced skin injury.

Regulatory cell death (RCD) is a type of cell death that depends on specific molecular mechanisms that are controllable and reversible [30]. Exploring the mechanisms of UVB-induced keratinocyte RCD has therapeutic potential for UVB-caused skin damage. The form of keratinocyte death induced by UVB has been most confirmed to be apoptosis for a long time [7]. Recent studies have found that a variety of RCDs other than apoptosis are involved in UVB-induced keratinocyte death, including GSDME-mediated pyroptosis [12, 31] and ferroptosis [9, 10]. In this study, we verified for the first time that UVB irradiation simultaneously upregulated indicators of pyroptosis, apoptosis and necroptosis in keratinocytes both in vivo and ex vivo, indicating that PANoptosis may be triggered in UVB-challenged keratinocytes. The biological effects observed in PANoptosis cannot be completely explained by pyroptosis, apoptosis, or necroptosis alone [16], and individual blockade of a key molecule of pyroptosis, apoptosis, or necroptosis does not prevent cell death [32]. To validate this feature of PANoptosis, we inhibited the downstream execution molecules of pyroptosis, apoptosis, and necroptosis by inhibiting GSDMD, caspase-3, and MLKL, respectively. The results showed that blocking any one of the above three RCDs alone was not sufficient to protect keratinocytes from UVB-induced cell death; only simultaneous blockade of all three RCDs by ZBP1 knockout can protect keratinocyte against cell death. Therefore, our study further confirmed the complexity of keratinocyte RCDs after UVB irradiation, including GSDME-mediated pyroptosis [12, 31], ferroptosis [9, 10], and PANoptosis, among which PANoptosis is the key lethal modality leading to keratinocyte death.

After sensing pathogen components, sensor proteins mediate the assembly of other proteins into a PANoptosome complex, which induces PANoptosis [17]. The proteins that constitute PANoptosome include three types: (1) PAMPs or DAMPs sensors, such as ZBP1, AIM2, NLRP3, and NLRP12; (2) adapters, such as FADD and ASC; (3) catalytic effectors, such as RIPK1, RIPK3, caspase-1, and caspase-8 [28, 29]. Most of the above components were elevated in PANoptosome-related studies during PANoptosis activation [29, 33–35]. In this study, we found that the protein levels of ZBP1, NLRP3, NLRP12, FADD, RIPK1, RIPK3, cleaved caspase-1, and caspase-8 increased in mice epidermis after UVB irradiation, while the expressions of AIM2 and ASC decreased. AIM2 is a common sensor that initiates inflammasomes, whereas ASC, as an adapter, is involved in multiple inflammasomes such as AIM2-, NLRP3-, and NLRP12-inflammasomes [29, 36]. It has been proved that AIM2 forms a complex with Pyrin and ZBP1 to drive PANoptosis in human THP-1 cells during HSV infection, in which AIM2 expression is also reduced [33]. Therefore, we speculate that the reduction in AIM2 and ASC levels may be due to the depletion of PANoptosome assembly in keratinocytes after UVB irradiation. Our results suggest that all of the above proteins are involved in the assembly of PANoptosome in UVB-irradiated mouse keratinocytes, but which molecules are essential in this process remain to be further elucidated by protein interaction analysis.

Our study demonstrated that ZBP1 was highly expressed in human sun-exposed skin, and ZBP1 knockout reduced keratinocyte death along with the expression of N-GSDMD, cleaved PARP, cleaved caspase-3 and p-MLKL, suggesting that ZBP1 is a key molecule in UVB-induced keratinocyte PANoptosis. However, we did not delve into how ZBP1 is regulated and initiates PANoptosome assembly in keratinocytes after UVB irradiation in this study. ZBP1, also known as DNA-dependent activator of IRFs (DAI), was originally found to be strongly induced by IFN-γ or lipopolysaccharide (LPS) and to play an important role in DNA-mediated activation of innate immunity [37]. UVB irradiation can induce the secretion of IFN-γ in keratinocytes [4]. Our group recently confirmed that autophagy-related gene 5 (ATG5) exacerbates UVB-induced keratinocyte ferroptosis and contributes to IFN-γ secretion and M1 polarization [10], which may lead to ZBP1 activation. The N-terminal of ZBP1 contains two Zα domains, which act as sensors to bind Z-DNA or Z-RNA and further recruit RIPK1, RIPK3, and TRIF through RIP homotypic interaction motif (RHIM) domains to induce various RCDs [38–41]. For a long time, researches have focused on the role of ZBP1 in sensing nucleic acid during cell death induced by bacterial, virus, or fungi infections [40, 42–45]. However, keratinocytes are in a non-infectious environment during UVB irradiation. Recent studies found that ZBP1 recognition of endogenous retroelements (EREs) - derived dsRNA triggered cell death and skin inflammation in RIPK1-deficient mice [46, 47]. Furthermore, impaired mitochondrial DNA (mtDNA) is another endogenous ligand that activates ZBP1 [40, 48]. It has been confirmed that UVB-induced impairment of mitophagy contributes to the increased cytoplasmic leakage of mtDNA in HaCaT cells [11]. Therefore, we speculate that ZBP1 may be triggered by IFN-γ stimulation, endogenous nucleic acid, or mtDNA in keratinocytes after UVB irradiation. More experiments are still needed to verify our speculation.

Previously, ZBP1-mediated PANoptosis has been proven to play a vital role in many infectious diseases by promoting cell death and inflammation to activate host defense or inflammatory tissue damage, such as C. albicans, A. fumigatus [44], SARS-CoV-2 [49, 50], influenza A virus [51, 52] and Yersinia infections [34]. We found that inhibiting ZBP1-mediated PANoptosis by ZBP1 knockout reduced UVB-induced keratinocyte death and epidermal thickening. This finding indicates that ZBP1-mediated PANoptosis is crucial to UVB-induced keratinocyte death. However, our results showed that inhibiting ZBP1-mediated PANoptosis had little effect on immune cell recruitment and inflammation. In other inflammatory skin diseases such as atopic dermatitis [20, 53, 54], effector molecules such as DAMPs or cytokines released by keratinocyte RCD act as cyclic amplifiers of immune cell-induced inflammation. We hypothesized that, unlike atopic dermatitis, the effects of keratinocyte PANoptosis in acute UVB-induced skin injury may be limited to mediating keratinocyte death, yet interplay with immune cells may not be significant. Moreover, Vats et al. reported that the inhibition of ferroptosis blocked necroinflammation in UVB-irradiated mice skin by preventing the release of HMGB1, so they suggested that acute UVB-induced skin inflammation is predominantly driven by keratinocyte ferroptosis [9]. This may also explain why the suppression of PANoptosis did not relieve UVB-induced acute skin inflammation in our study, as keratinocyte ferroptosis was not prevented. Although our current research has shown that inhibiting keratinocyte PANoptosis has little effect on UVB-caused acute inflammation, given that PANoptosis is also present in keratinocytes of human skin chronically exposed to UV, more research is still needed to completely understand the role PANoptosis plays in UV-caused chronic skin damage and inflammation.

Overall, this study demonstrated that UVB induces ZBP1-mediated PANoptosis in keratinocytes, which plays a crucial lethal role among diverse keratinocyte death modalities in UVB-induced skin injury. Individually inhibiting GSDMD-mediated pyroptosis, caspase-3-mediated apoptosis, or MLKL-mediated necroptosis is not sufficient to protect keratinocytes from UVB-induced cell death. UVB-induced keratinocyte death is rescued only when three RCDs are all inhibited by blockade of PANoptosome sensor ZBP1. However, we did not elucidate the precise mechanisms of how ZBP1 initiates and assembles PANoptosome in keratinocytes after acute UVB exposure, nor did we investigate the role of keratinocyte PANoptosis in UV-caused chronic skin damage, which will continue to be explored in the future. This study provides further evidence of the diversity of keratinocyte death fates after UVB irradiation and is instructive for the development of therapeutic strategies for UV-related skin diseases.

## Supplementary information

Figure S1

Figure S2

Supplementary Figure Legends

Original western blots

## Data Availability

All data presented in this manuscript are available upon request from corresponding authors.
