# Supplementary figures and images for: ZBP1-mediated PANoptosis is a crucial lethal form in diverse keratinocyte death modalities in UVB-induced skin injury
Source: Cell Death Dis. 2025 Jan 26;16(1):44. doi: 10.1038/s41419-025-07351-3 (PMC11762280; doi:10.1038/s41419-025-07351-3)

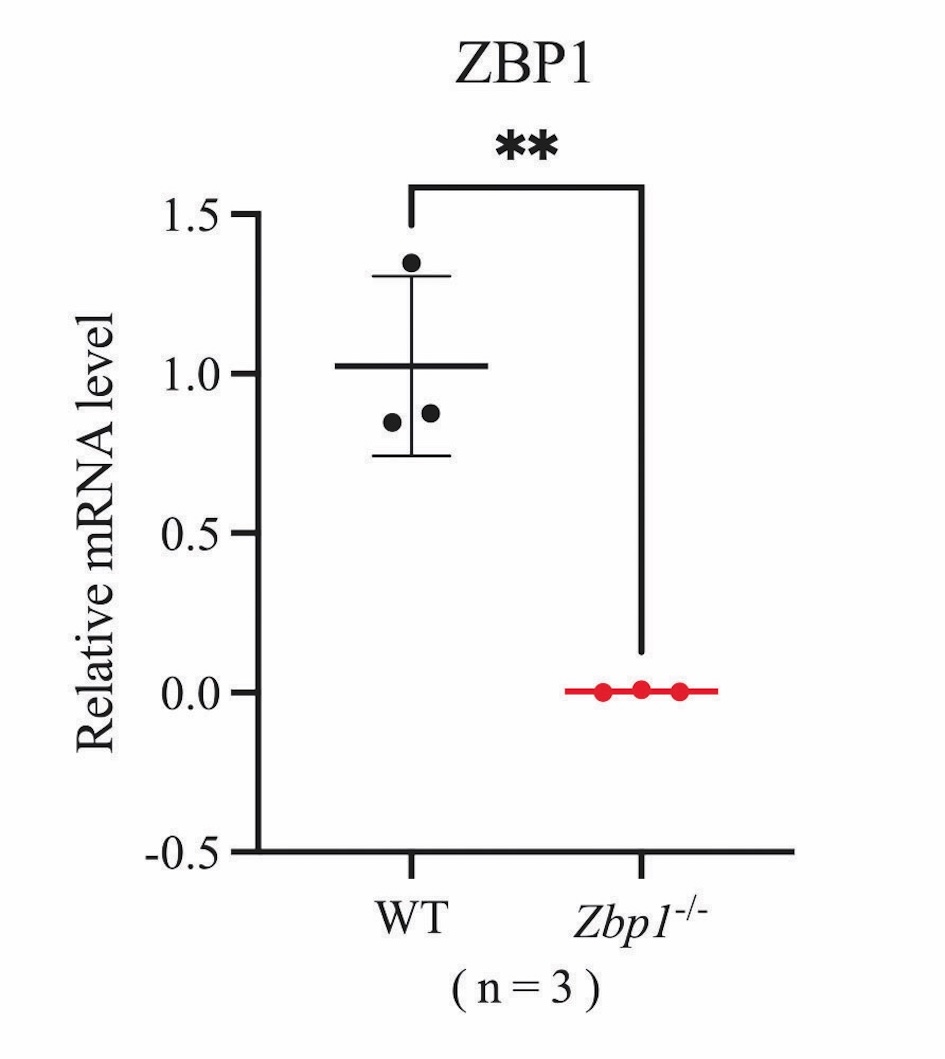

Supplement: Supplementary file 1 — Figure S1 [file 41419_2025_7351_MOESM1_ESM.jpg]

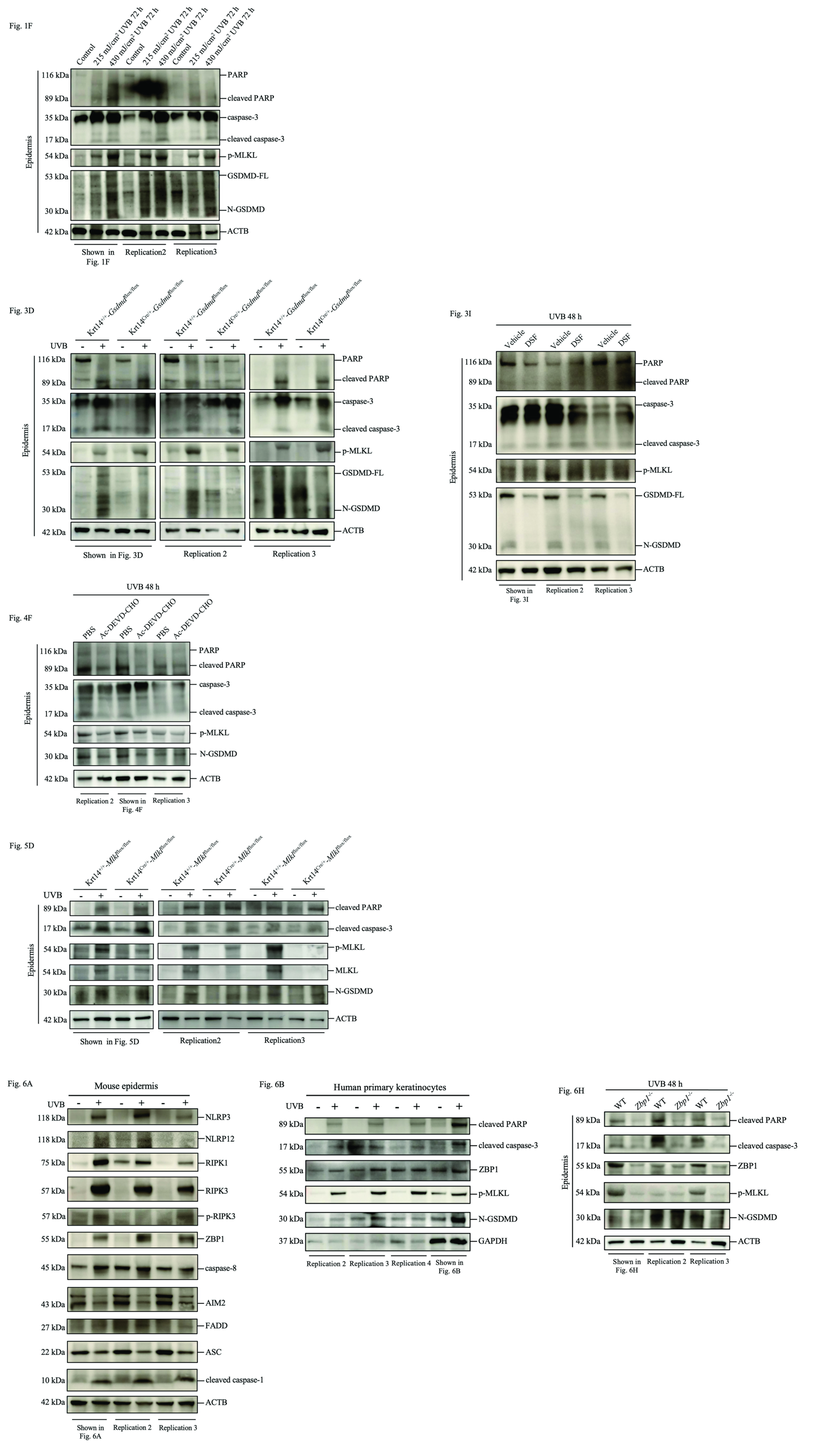

Supplement: Supplementary file 2 — Figure S2 [file 41419_2025_7351_MOESM2_ESM.jpg]

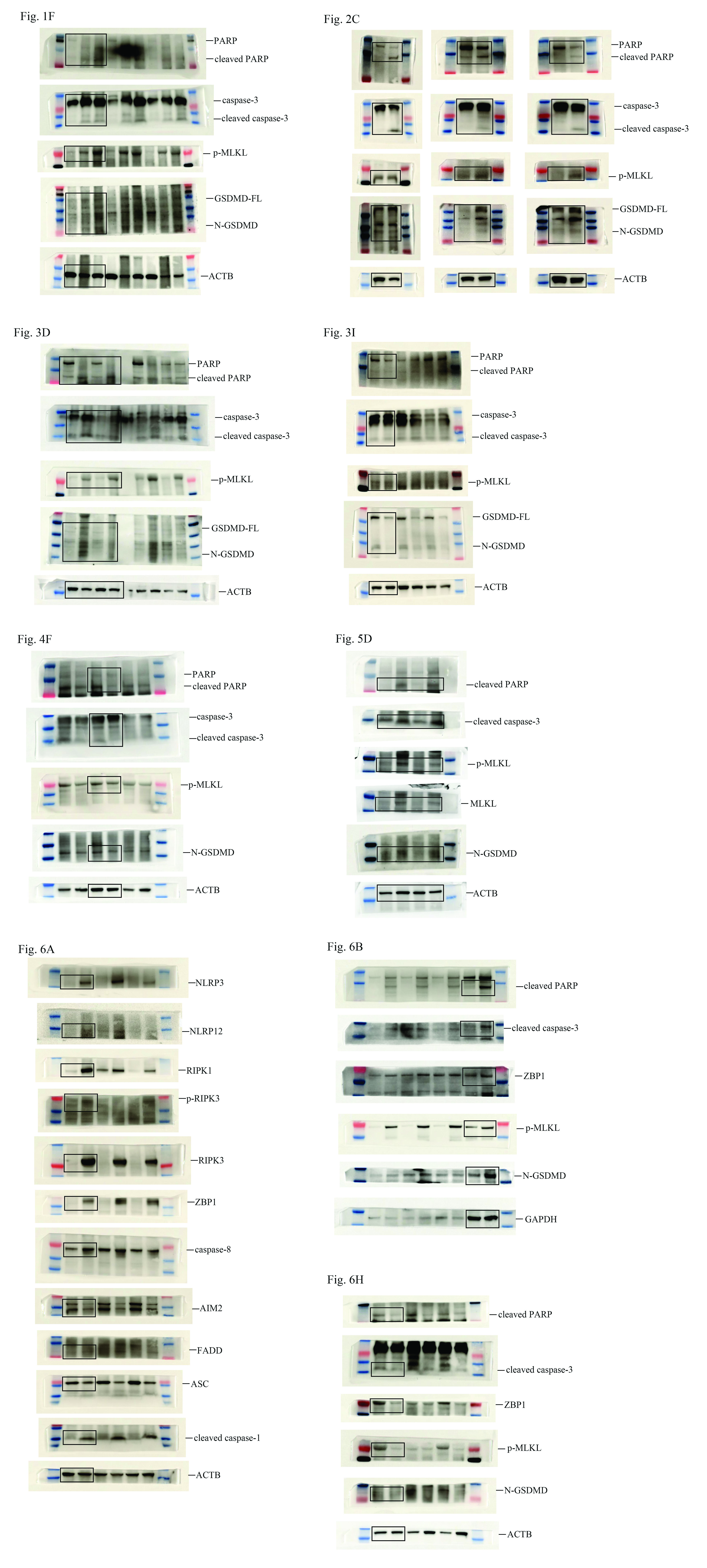

Supplement: Supplementary file 4 — Original western blots [file 41419_2025_7351_MOESM4_ESM.jpg]
