# Supplementary material for: ZBP1-mediated PANoptosis is a crucial lethal form in diverse keratinocyte death modalities in UVB-induced skin injury
Source: Cell Death Dis. 2025 Jan 26;16(1):44. doi: 10.1038/s41419-025-07351-3 (PMC11762280; doi:10.1038/s41419-025-07351-3)
Supplement: Supplementary file 3 — Supplementary Figure Legends [file 41419_2025_7351_MOESM3_ESM.docx]

**Supplementary Figure Legends**

**Fig. S1 The relative mRNA level of ZBP1 in WT and *Zbp1*^-/-^ mice after UVB irradiation by qRT-PCR.** (n = 3). Actin served as the reference gene. **P<0.01.

**Fig. S2 Replications of western blotting bands.**
